# The LDHC-STAT3 Signaling Network Is a Key Regulator of Basal-like Breast Cancer Cell Survival

**DOI:** 10.3390/cancers16132451

**Published:** 2024-07-04

**Authors:** Adviti Naik, Remy Thomas, Martin Sikhondze, Abeer Babiker, Boucif Lattab, Hanan Qasem, Umar Jafar, Julie Decock

**Affiliations:** 1 Cancer Research Center, Qatar Biomedical Research Institute (QBRI), Hamad Bin Khalifa University (HBKU), Qatar Foundation (QF), Doha P.O. Box 34110, Qatara.babiker2201@hotmail.com (A.B.); haqa24936@ hbku.edu.qa (H.Q.); umja44783@hbku.edu.qa (U.J.); 2 College of Health and Life Sciences (CHLS), Hamad Bin Khalifa University (HBKU), Qatar Foundation (QF), Doha P.O. Box 34110, Qatar

**Keywords:** LDHC, STAT3, DNA damage repair, cell survival, basal-like breast cancer

## Abstract

**Simple Summary:**

Breast cancer remains the deadliest cancer among women worldwide. A major challenge in breast cancer care lies in the identification of drug targets that enable the specific killing of tumor cells without affecting normal cells. Previously, we showed that lactate dehydrogenase C (LDHC) plays an important role in regulating tumor genomic integrity and that targeting LDHC greatly reduces breast tumor cell survival and improves the efficacy of common anti-cancer drugs. The aim of our study was to gain insight into the molecular pathways that are associated with LDHC in tumors. Using three breast cancer cell lines, we found that the effect of LDHC targeting on tumor cell survival was negatively impacted by the activation of the STAT3 pathway in some cell lines and could be restored by STAT3 inhibition. Thus, LDHC is a viable target for breast cancer treatment in a subtype-specific manner.

**Abstract:**

Breast cancer treatment has evolved drastically with the addition of immunotherapy and novel targeted drugs to the current treatment options. However, achieving long-term responses with minimal adverse events remains challenging. Cancer testis antigens (CTAs) offer novel opportunities for drug development thanks to their tumor specificity, immunogenicity, pro-tumorigenic functions, and negative prognostic connotations. We previously reported that lactate dehydrogenase C (LDHC) plays a key role in regulating genomic stability and that targeting LDHC significantly improved treatment response to DNA damage response drugs in breast cancer. Here, we explored the molecular mechanisms associated with *LDHC* silencing in two basal-like breast cancer cell lines, MDA-MB-468 and BT-549, and a Her2-enriched breast cancer cell line, HCC-1954. Transcriptomic analyses identified the cell line-dependent differential activation of the pro-survival STAT3 pathway following LDHC depletion. While *LDHC* silencing significantly compromised cell survival in basal-like breast cancer cells in conjunction with a downregulation of STAT3 signaling, the opposite effect was observed in Her2-enriched breast cancer cells, which demonstrated the enhanced activation of the pro-survival STAT3 signaling pathway. The inhibition of STAT3 not only reversed the unfavorable effect of *LDHC* silencing in the Her2-enriched cancer cells but also demonstrated significant anti-cancer activity when used as a single agent. Our findings suggest that the LDHC-STAT3 signaling axis plays a role in regulating breast tumor cell survival in a subtype-dependent manner. Thus, LDHC-targeted therapy could be a viable therapeutic approach for a subset of breast cancer patients, particularly patients with basal-like breast cancer, whereas patients carrying Her2-enriched tumors may likely benefit more from monotherapy with STAT3 inhibitors.

## 1. Introduction

Breast cancer is still the most commonly diagnosed and deadliest cancer in women worldwide despite many significant advances in cancer care over the last few decades [1]. Tremendous strides have been made in breast cancer treatment with the addition of targeted therapy and immunotherapy to complement conventional surgery, chemotherapy, and radiation. However, one of the major challenges remains the identification of tumor-specific targets that can induce durable responses with minimal adverse side effects.

Cancer testis antigens (CTAs) form a group of tumor-associated antigens that display a restricted expression pattern in normal somatic tissues and aberrant expression in a wide range of cancers including breast cancer [2,3,4]. In addition, they promote multiple cancer hallmarks and demonstrate high immunogenicity in different tumor types, as exemplified by NY-ESO-1, MAGE-A3 and PRAME [5,6,7]. This feature positions CTAs as promising candidates for therapeutic targeting, facilitating cancer therapy with minimal adverse side effects in contrast to many existing treatment modalities. For instance, the targeting of CTAs such as NY-ESO-1 and PRAME has shown notable pre-clinical anti-tumor activity and is currently undergoing evaluation in clinical trials using vaccine-based or adoptive cell therapy [6,7,8]. Lactate dehydrogenase C (LDHC) is a CTA that has recently gained interest thanks to its highly restricted expression pattern, pro-tumorigenic functions, immunogenic properties and association with unfavorable outcomes in solid tumors [9,10,11,12,13,14,15]. In normal physiological conditions, LDHC expression is solely observed in the testis, particularly in adult spermatocytes, where it regulates sperm energy metabolism, thereby affecting sperm capacitation, motility, and fertilizing capacity [16]. LDHC expression can be observed in various tumor types following transcriptional de-repression; however, its biological functions in cancer remain largely unknown. We previously demonstrated the aberrant expression of *LDHC* in breast tumor tissues of the TCGA breast cancer dataset, in particular in basal-like and Her2-enriched breast tumors, which was associated with worse overall and disease-specific survival [11].

Few studies have shown that LDHC expression enhances tumor cell migration and invasion and promotes xenograft tumor growth and metastatic propensity [12,13,17]. Mechanistically, LDHC was found to induce the expression of proteins involved in the regulation of cell proliferation (cyclin D1, c-Myc) and epithelial-to-mesenchymal-transition (E-Cadherin, N-Cadherin, vimentin, Twist, Slug, Snail, MMP-2, MMP-9) through the likely activation of the PI3K/Akt/GSK-3β signaling pathway [12,13]. We recently demonstrated that LDHC plays a vital role in regulating and maintaining tumor cell genomic integrity, suggesting that targeting LDHC could present a novel opportunity for therapeutic intervention [11]. We found that the stable silencing of *LDHC* drastically compromised breast cancer cell survival through the accumulation of DNA damage, microtubule destabilization and cell cycle dysregulation, resulting in enhanced cell death, mitotic catastrophe, and senescence. Moreover, *LDHC* silencing improved treatment response to DNA damage repair-related drugs such as cisplatin and olaparib, underscoring the potential role of LDHC as a therapeutic target. As such, LDHC-based interventions could be administered as monotherapy or in combination with common anti-cancer drugs to target tumor cells in a highly specific manner while minimizing adverse side effects.

In this study, we sought to gain more insight into the effect of LDHC targeting on the transcriptomic landscape of cancer cells, particularly in cancer cells of the basal-like and Her2-enriched breast cancer subtypes, which demonstrate a higher expression of LDHC in corresponding human tumor tissues [10]. Using multiple breast cancer cell line models (MDA-MB-468, BT-549, HCC-1954), we identified STAT-3 signaling to be a cell line-dependent factor determining cancer cell survival following *LDHC* silencing. While *LDHC* silencing greatly reduced the cell survival of basal-like breast cancer cells via the downregulation of STAT3 signaling, it exerted the opposite effect in Her2-enriched breast cancer cells, which could be reversed by STAT3 inhibition. Furthermore, the treatment of Her2-enriched breast cancer cells with a STAT3 inhibitor alone demonstrated a significant reduction in cancer cell survival, suggesting a likely benefit of STAT3 inhibitor monotherapy for this subset of patients, whereas basal-like breast cancer patients would possibly benefit from LDHC-targeted therapy.

## 2. Materials and Methods

### 2.1. Cell Culture

MDA-MB-468, HCC-1954 and BT-549 cell lines were purchased from the American Tissue Culture Collection (ATCC, Manassas, VA, USA). MDA-MB-468 and HCC-1954 cells were maintained in Dulbecco’s Modified Eagle’s Medium (Gibco-BRL, Gaithersburg, MD, USA) supplemented with 10% (*v*/*v*) Fetal Bovine Serum (Hyclone US origin, GE Lifescience, Milwaukee, WI, USA), 50 U/mL of penicillin, and 50 μg/mL of streptomycin (Gibco-BRL). BT-549 cells were maintained in ATCC-formulated Roswell Park Memorial Institute 1640 medium (Gibco-BRL) supplemented with 10% (*v*/*v*) Fetal Bovine Serum (Hyclone US origin, GE Lifescience), 50 U/mL of penicillin, 50 μg/mL of streptomycin (Gibco-BRL), and 0.023 IU/mL of insulin (Sigma-Aldrich, St. Louis, MO, USA). All cell lines were maintained in a humidified incubator at 37 °C and 5% CO_2_, and regular mycoplasma testing was performed using a PCR-based assay.

### 2.2. LDHC Silencing

Stably silenced *LDHC* MDA-MB-468 cells were established as previously described [11]. The transient silencing of *LDHC* in BT-549 and HCC-1954 was achieved using LDHC siRNA #1–4 smartpool (siGENOME SMARTpool) and control siRNA #1–4 smartpool (siGENOME Non-Targeting siRNA Pool#1, D-001206-13-20) from Dharmacon (Lafayette, CO, USA). Adherent cells were transfected at 60–70% confluency with 20 nM of siRNA #1–4 and Lipofectamine RNAiMAX (Thermo Fisher Scientific, Walthan, MA, USA) following the manufacturer’s instructions. The silencing of *LDHC* was assessed by real time qRT-PCR and Western blotting.

### 2.3. RNA Extraction and Quality Assessment

Total RNA was isolated 24 h after transient silencing using the PureLink RNA Mini kit (Ambion, Austin, TX, USA) following the manufacturer’s protocol. The RNA quantity and purity were assessed by Nanodrop measurement. The reverse transcription of 1 µg of RNA was performed using MMLV-Superscript and random hexamers, resulting in a final concentration of 50 ng/µL of cDNA.

### 2.4. Quantitative Real-Time Reverse Transcription Polymerase Chain Reaction

Real-time PCR to assess *LDHC* expression was conducted using specific 5′FAM-3′MGB TaqMan gene expression primer/probe sets (Hs00255650_m1, Applied Biosystems, Foster City, CA, USA). The expression of *STAT3* (F: TCAAGCAGTTTCTTCAGAGCAG, R: AAGGCGTGATTCTTCCCACA) and *PRKCD* (F: TTCTTTGGGCAACCCACCTT, R: CAGCGTTACATTGCCTGCAT) was quantified using primers for SYBR-based qPCR and designed using PrimerBLAST (NCBI, Bethesda, MD, USA) and the PowerUp SYBR Green master mix (Applied Biosystems). A total of 100 ng of cDNA was used per reaction. The QuantStudio 7 Real-time PCR instrument (Applied Biosystems, USA) was used to conduct the qRT-PCR. The specificity of SYBR-based PCR reactions was verified using melt curve analysis. Each real-time PCR reaction was performed in duplicate, and a no template control (NTC) was performed to identify non-specific amplification for each primer pair tested in all experimental runs. Expression levels were normalized to the housekeeping gene *RPLPO* (TaqMan primer/probe 4333761F or SYBR primers F: TCCTCGTGGAAGTGACATCG, R: TGGATGATCTTAAGGAAGTAGTTGG). Differential gene expression was calculated using the 2^−ΔΔCt^ method. Primers were designed using PrimerBLAST with the following parameters: amplicon size of 70–200 bp; GC content of 50–60%; primer melting temperature between 57 °C and 63 °C, optimally 60 °C; and primers spanning exon–exon junctions. All other parameters were set as default.

### 2.5. RNA Sequencing

Total RNA was depleted from ribosomal RNA and random-primed for cDNA synthesis using the TruSeq stranded total RNA kit (Illumina, San Diego, CA, USA). RNA sequencing was performed on the Illumina HiSeq2500 platform (Illumina) with Paired End 25× coverage (PE100-125). The FASTQ files were trimmed to remove adaptor sequences using flexbar (v3.0.3) and aligned to the GRCh37/hg19 reference genome using hisat2 (v2.0.5), resulting in an average of 10–15 M aligned reads. Reads were counted to genomic features using subreads (v1.5.5). RNA-seq data were corrected for GC content and normalized within and between lanes using the R package EDASeq (v2.12.0), and they were quantile-normalized using preprocessCore (v1.36.0). Volcano plots and heatmaps of differentially expressed genes were generated using the EnhancedVolcano (v 1.12.0), ggplot2 (v 3.4.1) and ComplexHeatmap (v 2.10.1) R packages. Differentially expressed genes associated with *LDHC* silencing (*p* ≤ 0.05) were subjected to Ingenuity Pathways Analysis (IPA, Qiagen, The Netherlands) to map cellular functions, canonical pathways, molecular networks, and upstream regulators. 

### 2.6. Western Blotting

After 48 h of *LDHC* silencing, cell protein lysate was isolated using RIPA buffer (Pierce, Waltham, MA, USA) supplemented with a HALT protease and phosphatase inhibitor cocktail (Thermo Fisher Scientific). Western blotting was performed using a standard protocol as previously described [18]. The primary antibodies used included anti-LDHC (Abcam, Cambridge, UK, #ab52747, 1:500), anti-STAT3 (Cell Signaling, Danvers, MA, USA, #4904, 1:1000), anti phospho S727-STAT3 (Cell Signaling, #34911, 1:1000), anti-PKC delta (Abcam, #ab182126, 1:1000), anti phospho S645-PKC delta (Abcam, #ab108972, 1:1000), anti phospho S139-γH2AX (Abcam, #ab11174, 1:1000), anti-PARP (Cell Signaling, #9542, 1:1000), and rabbit anti-βactin (Cell Signaling, #4970, 1:1000). Horseradish peroxidase (HRP)-linked anti-rabbit/mouse secondary antibody incubation followed by enhanced chemiluminescent substrate (ECL) Supersignal West Femto (Pierce) incubation was used to visualize the protein bands of interest on the ChemiDoc XRS+ Imaging system (Biorad, Hercules, CA, USA). Images acquisition and densitometry analysis were performed using the Image Lab software version 6.1.0 (Biorad).

### 2.7. Clonogenic Assay

HCC-1954 cells were seeded at 5 × 10^3^ cells/well in twelve-well plates and maintained in complete growth media for 7 days, after which the cells were transfected with either *LDHC* or non-targeting control (CTRL) siRNA for 24 h. After 7 days, the cells were washed with PBS and stained with crystal violet (5% crystal violet, 25% methanol) for 20 min at 37 °C. Excess stain was washed away with distilled water, and images were recorded. Next, the stain was solubilized using 10% acetic acid for 2 h and transferred into a clean transparent 96-well plate, and absorbance was measured at 590 nm.

### 2.8. CellTiter-Glo Cell Viability Assay

Cells were harvested by centrifugation and plated at 10^5^ cells/well in an opaque 96-well plate using complete growth media. Cell lysis was induced by adding an equivalent volume of CellTiter-Glo^®^ reagent (Promega, Madison, WI, USA), after which the plate was transferred to an orbital shaker for 2 min. After 10 min of incubation at room temperature, luminescence was recorded using the Promega GloMax^®^-Multi Detection system.

### 2.9. Annexin V/PI Flow Cytometry

Cells were harvested by centrifugation, washed in phosphate-buffered saline (PBS), and resuspended in 1× Annexin-binding buffer (Thermo Fisher Scientific). Cells were stained with Annexin V BV421 (BD Biosciences, Franklin Lakes, NJ, USA) and propidium iodide (PI) at room temperature for 15 min. Flow cytometry analysis was performed using the LSRFortessa X-20 system (BD Biosciences) and flowjo software (v10.8.1).

### 2.10. STAT3 Inhibition

HCC-1954 cells were seeded at 2 × 10^5^ cells/well in six-well plates and transfected with either *LDHC* or CTRL siRNA for 24 h. Next, transfected cells were treated with 138.5 µM (IC50) of STAT3 inhibitor VI (Millipore, Burlington, MA, USA) or DMSO vehicle control. After 72 h, cells were used for Western blot analyses, the CellTiter Glo viability assay, a clonogenic assay and Annexin V/PI flow cytometry.

### 2.11. Statistical Analyses

Data normality was assessed using the Shapiro–Wilk test, and statistical analyses were performed using unpaired two-tailed Student’s *t*-test. A *p*-value of≤ 0.05 was defined as statistically significant. Data are represented as the mean  ±  standard error of mean (SEM) of at least three independent biological replicates. Statistical analyses and data representation were performed using GraphPad Prism v8.0.0.

## 3. Results

### 3.1. Depletion of LDHC in MDA-MB-468 Cells Induces Transcriptomic Changes That Affect Cell Cycle Progression and Tumor Cell Survival

**Figure 1:**
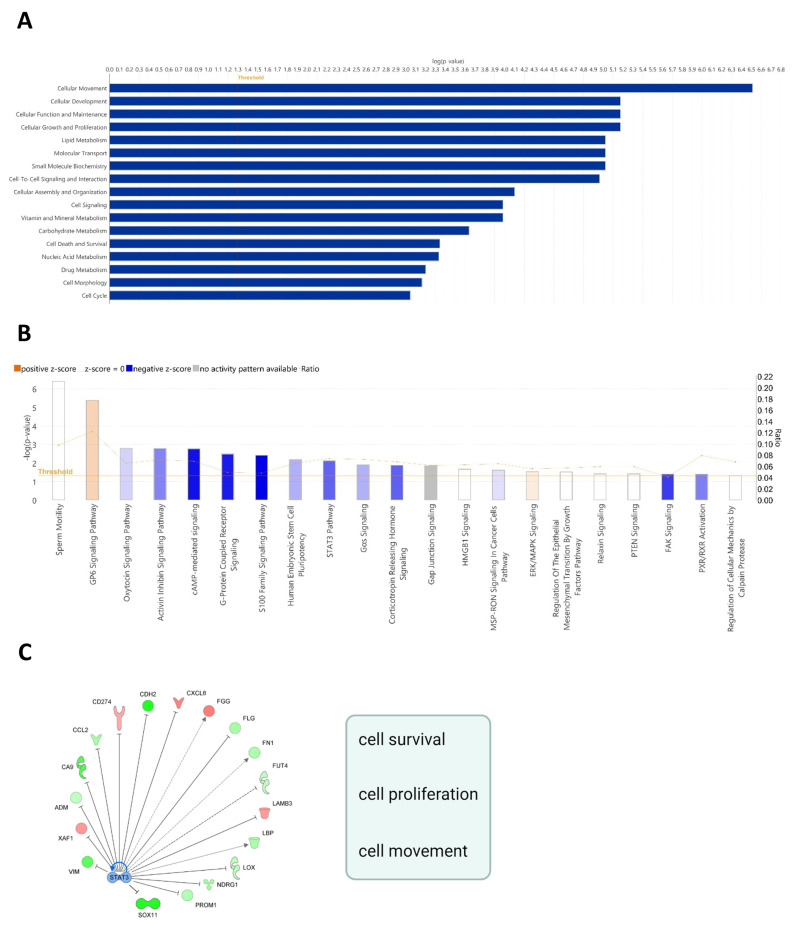
Cellular function, pathway, and network analysis of basal-like MDA-MB-468 cells with a compromised survival phenotype following *LDHC* knockdown. (**A**) Bar graph depicting cellular and molecular functions associated with differentially expressed genes following *LDHC* silencing. (**B**) Canonical pathways associated with *LDHC* silencing. Positive z-scores (orange) represent net activated pathways, and negative z-scores refer to inhibited pathways. (**C**) Illustration of STAT3 mechanistic network with the activation status of the upstream regulators of differentially expressed genes. Up- and down-regulated genes are depicted in red and green, respectively; predicted STAT3-mediated activation and inhibition are shown by arrow heads and flat caps, respectively; and dashed lines represent conflicting or inconsistent relations.

We previously demonstrated that the stable silencing of *LDHC* dysregulates the DNA damage response and affects the mitotic fidelity and long-term survival of breast cancer cells, particularly in basal-like breast cancer cell lines [11]. These findings prompted us to further investigate the molecular alterations and network perturbations associated with *LDHC* silencing, focusing on the LDHC loss-of-function MDA-MB-468 cell line model that previously demonstrated a robust cellular phenotype. Ingenuity Pathway Analysis (IPA) revealed that the differentially expressed genes associated with *LDHC* silencing were enriched in cellular functions related to cellular movement, cellular development, cellular function and maintenance, cellular growth and proliferation, cellular signaling, metabolism, cell death and survival, and the cell cycle (Figure 1A). Canonical pathway analysis (Figure 1B) identified a net inhibitory effect (z-score ≤ −1) on multiple signaling pathways, with the top five most significantly dysregulated cancer-related pathways being activin inhibin signaling (*p* = 0.002, z = −1.3), cAMP-mediated signaling (*p* = 0.002, z = −2.5), G protein-coupled receptor signaling (*p* = 0.003, z = −2.4), the S100 family signaling pathway (*p* = 0.004, z = −2.7) and the STAT3 pathway (*p* = 0.008, z = −1.6). Looking at the ratio of molecules affected within each of these pathways, we found that *LDHC* silencing extensively impacted the STAT3 pathway, with the expression of ten molecules in the pathway being altered (10/135). Furthermore, network analysis indicated that STAT3 is an upstream regulator of several molecules that are involved in cell survival, proliferation and cell movement (Figure 1C). Collectively, these analyses corroborate our previous findings of dysregulated cell cycle progression and reduced long-term cancer cell survival following *LDHC* silencing [11].

### 3.2. LDHC Silencing Perturbs STAT3 Pro-Oncogenic Signaling in a Cell Line-Dependent Manner

**Figure 2:**
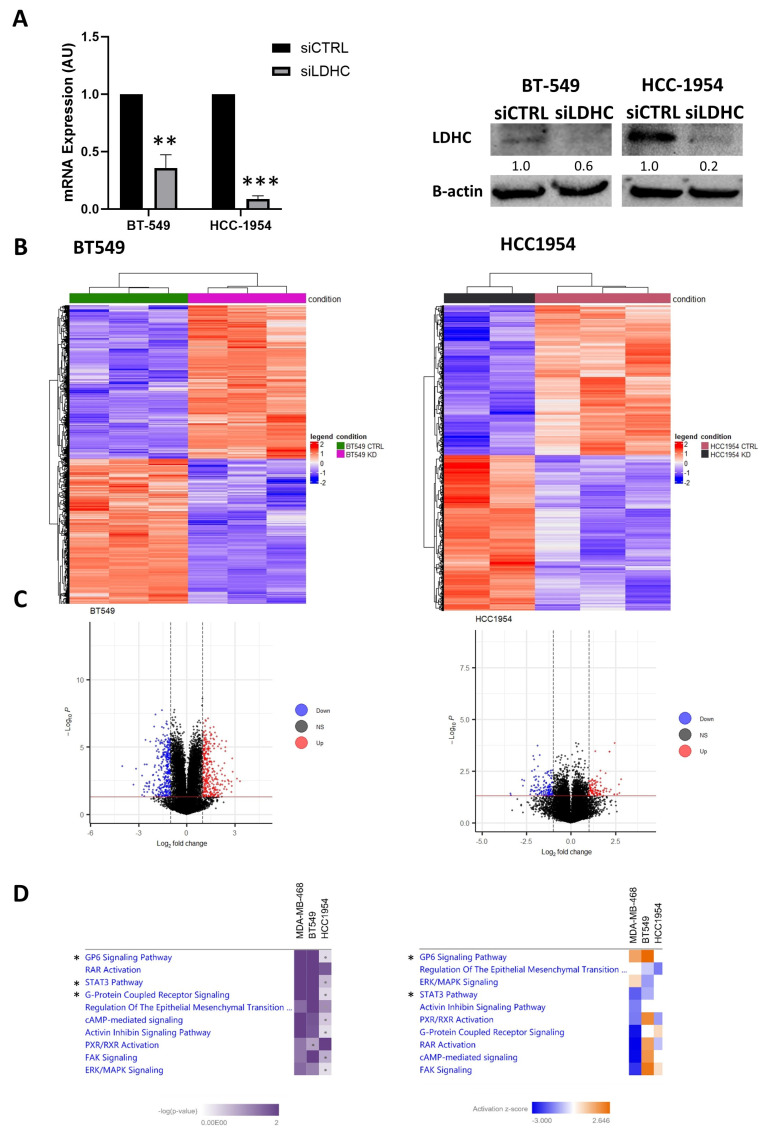
Transcriptomic analysis of *LDHC*-silenced breast cancer cell line models. (**A**) qRT-PCR and Western blotting results demonstrate efficient *LDHC* silencing in BT-549 and HCC-1954 cells. Bar charts indicate the mean with standard error (±SEM) of three biological replicates. Blots are representative of three independent biological replicates. *RPLPO* was used to normalize *LDHC* mRNA expression, and β-actin served as the Western blot loading control. ** *p* ≤ 0.01, *** *p* ≤ 0.001. (**B**) Heatmap of differentially expressed genes in BT-549 and HCC-1954 cells following *LDHC* silencing. (**C**) Volcano plot depicting the significant upregulated (red) or downregulated (blue) genes upon LDHC knockdown (log2FC > 1, *p* < 0.05). (**D**) Comparative canonical pathway analysis of LDHC-silenced MDA-MB-468, BT-549 and HCC-1954 cells. The left heatmap depicts the significance of the altered pathways, and the right heatmap depicts the activation scores of the significantly altered pathways. * indicates the top three most significantly dysregulated pathways in both MDA-MB-468 and BT-549 but not in the HCC-1954 cell line. The uncropped blots are shown in File S1.

Next, we sought to validate our findings in one additional basal-like breast cancer cell line, BT-549, and investigated whether *LDHC* silencing differentially affects gene expression in other breast cancer subtypes through a comparative analysis of the Her2-enriched HCC-1954 cell line. The efficient knockdown of LDHC expression was achieved in both cell lines, which were subsequently subjected to transcriptomic analysis (Figure 2A and Figure S1A). The hierarchical clustering of differentially expressed genes revealed distinct transcriptomic profiles between *LDHC*-silenced and control cells (Figure 2B). *LDHC* silencing in BT-549 cells resulted in the significant upregulation of 309 genes and the significant downregulation of 319 genes (abs log2 FC ≥ 1, *p* ≤ 0.05), as illustrated in the volcano plot (Figure 2C and Table S1). On the other hand, in HCC-1954, we identified 114 upregulated genes and 139 downregulated genes (abs log2 FC ≥ 1, *p* ≤ 0.05) following the knockdown of LDHC expression (Figure 2C and Table S2). Comparative pathway analysis identified the GP6, STAT3 and G protein-coupled receptor signaling pathways to be the top three most significantly dysregulated pathways in both basal-like breast cancer cell lines, MDA-MB-468 and BT-549, but not in the Her2-enriched HCC-1954 cell line (Figure 2D—left). Looking at the directional effect of *LDHC* silencing, we found that reducing LDHC expression inhibited the STAT3 pathway and promoted the GP6 signaling pathway in both basal-like cell lines in contrast to the HCC-1954 cell line (Figure 2D—right).

### 3.3. Role of LDHC-STAT3 Molecular Axis in Tumor Cellular Fitness

**Figure 3:**
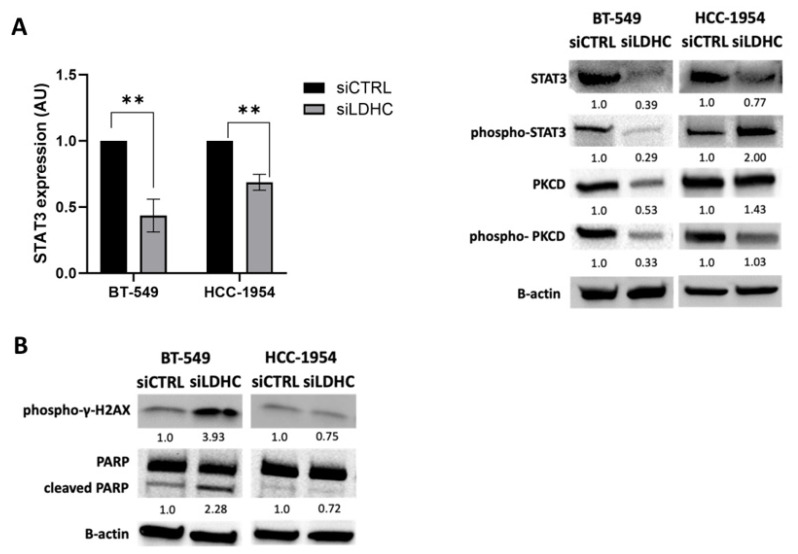
STAT3 signaling determines the cellular phenotype of LDHC-silenced BT-549 and HCC-1954 cells. (**A**) *STAT3* mRNA expression and Western blot of total and phosphorylated STAT3 and total and phosphorylated PKC delta protein expression. Bar charts indicate the mean with standard error (±SEM) of three biological replicates. Blots are representative of three independent biological replicates. *RPLPO* was used to normalize *STAT3* mRNA expression, and β-actin served as the Western blot loading control. ** *p* ≤ 0.01. (**B**) Western blot analysis of DNA damage marker phospho-γH2AX and apoptosis markers PARP and cleaved PARP. Blots are representative of three independent biological replicates, and β-actin was used as the protein loading control. The uncropped blots are shown in File S1.

To further decipher the differential role of STAT3 signaling in BT-549 and HCC-1954 *LDHC*-silenced cells, we investigated the expression and activation status of STAT3. In line with the transcriptomic analysis, we found that *LDHC* silencing negatively regulated STAT3 signaling in BT-549 but not in HCC-1954 cells, albeit with a reduction in STAT3 mRNA expression (Figure 3A—left). While the protein expression of total STAT3 and activated phospho-STAT3 levels were reduced in BT-549 *LDHC*-silenced cells, the latter was increased in HCC-1954 cells following *LDHC* silencing (Figure 3A—right and Figure S1B). In accordance, the protein expression of total and activated phospho-protein kinase C (PKC) delta, a primary regulator of STAT3 phosphorylation, was strongly reduced in BT-549 cells upon *LDHC* silencing, whereas the total PKCD, but not phospho-PKCD, expression was upregulated in HCC-1954 cells. Given the key role of STAT3 signaling in cancer cell proliferation and survival, the question of whether the cell line-dependent regulation of this pathway could result in a divergent effect of *LDHC* silencing on tumor cell survival arose. Analogously with our previous observations in MDA-MB-468 cells, we found that reducing LDHC expression in BT-549 cells increased the expression of the DNA damage marker phopsho-γ-H2AX and cleaved PARP, indicating that *LDHC* silencing induced tumor cell death (Figure 3B and Figure S1C). In contrast, the knockdown of *LDHC* in HCC-1954 cells did not induce the expression of cleaved PARP and slightly decreased phopsho-γ-H2AX expression. Together, these findings suggest that *LDHC* silencing reduces the cancer cell viability of basal-like breast cancer cells in part through the downregulation of STAT3 signaling, whereas in HCC-1954 cells, the activation of STAT3 (through unknown mechanisms) negates this biological process, highlighting the cell line-dependent effects of *LDHC* silencing.

### 3.4. STAT3 Inhibition Reduces Cell Viability in Tumor Cells That Are Resistant to LDHC Silencing

**Figure 4:**
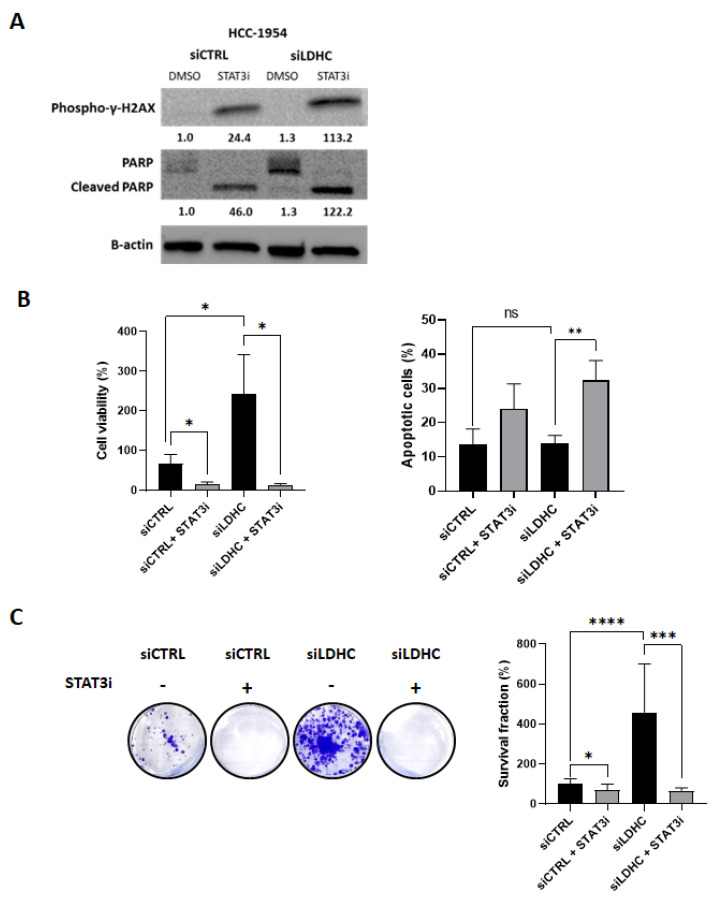
STAT3 activation in HCC-1954 cells impairs the survival phenotype associated with *LDHC* silencing. (**A**) Western blot of DNA damage and apoptosis markers following *LDHC* silencing in combination with STAT3 inhibition. Blots are representative of three independent biological replicates, and β-actin was used as the protein loading control. The numbers under each lane in the blot represent mean densitometry values (arbitrary units) normalized to β-actin from three independent experiments. (**B**) Cell viability (CellTiter-Glo assay) and apoptosis analyses (Annexin V/PI flow cytometry and late apoptotic cells) of *LDHC*-silenced HCC-1954 cells treated with the STAT3 inhibitor or vehicle control. Bar charts indicate the mean with standard error (±SEM) of three biological replicates. * *p* ≤ 0.05, ** *p* ≤ 0.01, ns non-significant. (**C**) Clonogenic assay of HCC-1954 cells after LDHC knockdown and STAT3 inhibition, with absorbance measurement of crystal violet staining. In the representative image of the clonogenic assay, the bar charts indicate the mean with standard error (±SEM) of three biological replicates. * *p* ≤ 0.05, *** *p* ≤ 0.001, **** *p* ≤ 0.0001. The uncropped blots are shown in File S1.

To further elucidate the role of STAT3 signaling in HCC-1954 cells, the cells were treated with a STAT3 inhibitor, and its effects on both short- and long-term survival were assessed. The inhibition of pro-oncogenic STAT3 signaling induced the expression of phospho-γ-H2AX and cleaved PARP in HCC-1954 cells (Figure 4A and Figure S1D). Notably, *LDHC*-silenced HCC-1954 cells demonstrated a higher cell viability than their control counterparts, likely resulting from the activation of the pro-oncogenic STAT3 signaling pathway, which could be reversed by STAT3 inhibition (Figure 4B—left). In line with this observation, STAT3 inhibition induced apoptotic cell death in both siCTRL and siLDHC cells (Figure 4B—right). Furthermore, the *LDHC* silencing of HCC-1954 cells was associated with a higher clonogenic ability that was significantly reduced by STAT3 inhibition (Figure 4C). Overall, STAT3 inhibition reversed the unfavorable effects of *LDHC* silencing in HCC-1954 cells and reduced the cancer cell survival of siCTRL cells, indicating that HCC-1954 cells do not benefit from LDHC-targeted therapy but may benefit from monotherapy with STAT3 inhibitors.

## 4. Discussion

Cancer treatment has seen a shift from a one-size-fits-all approach towards a more tailored, personalized approach, whereby tumor phenotypic and genotypic heterogeneity is taken into consideration. One of the major drivers behind this shift is the apparent difference in treatment responses and clinical outcomes between patients that are affected by the same cancer. The need for novel treatment approaches that allow a more individualized treatment plan comes with the task of the careful selection of tumor targets. The favorable characteristics of a novel tumor target would include highly specific tumor expression, biological function supporting tumor survival, and an inherent ability to induce an anti-tumor immune response. In this respect, we and others have shown that LDHC is expressed in various tumor types, can elicit anti-tumor cellular immune responses, is associated with worse clinical outcomes, and supports tumorigenesis and progression through multiple biological processes [9,10,11,12,13,14,15,17]. More specifically, we found that *LDHC* silencing greatly reduces tumor cellular fitness and the long-term survival of basal-like breast cancer cells [11]. Moreover, we demonstrated that reducing LDHC expression potentiates the efficacy of common anti-cancer drugs, illustrating the therapeutic potential of LDHC as a novel anti-cancer target for combination therapy in addition to single-agent therapy. 

Based on our previous findings, in the current study, we explored the molecular alterations associated with *LDHC* silencing to better understand the mechanistic basis of the observed cellular phenotype and to facilitate the identification of proxy synergistic targets. The comparative analysis of three breast cancer cell lines revealed cell line-dependent differences in the cellular phenotype and molecular landscape following *LDHC* silencing. 

**Figure 5:**
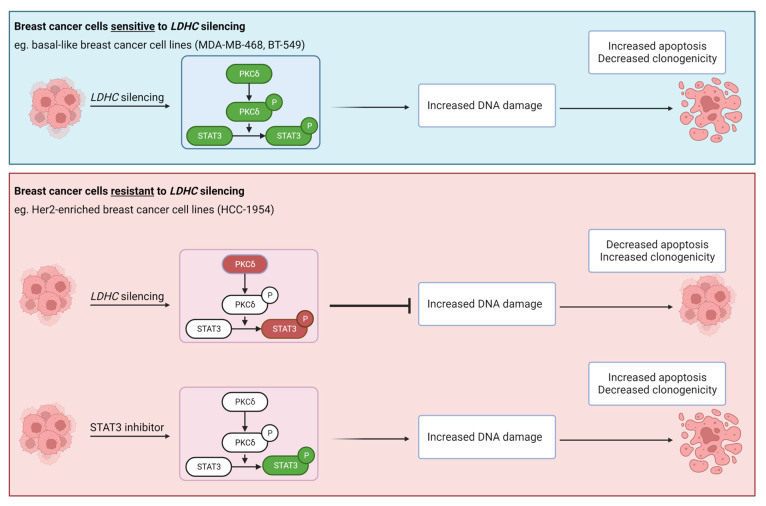
Schematic representation of LDHC-targeted therapy sensitivity and resistance. The targeting of LDHC in basal-like breast cancer cells induces excess DNA damage, leading to increased apoptosis and decreased clonogenic ability, and it is associated with the decreased expression of phospho-PKCD, total PKCD, phospho-STAT3 and total STAT3. In contrast, Her2-enriched breast cancer cells exhibit treatment resistance to LDHC targeting, associated with the enhanced activation of the pro-survival STAT3 pathway, and may benefit from monotherapy with STAT3 inhibitors. Molecules highlighted in green are downregulated, while red molecules are upregulated.

In contrast to what we observed in basal-like breast cancer cells, the depletion of LDHC in the Her2-enriched breast cancer cell line HCC-1954 did not induce DNA damage or reduce short- or long-term cancer cell survival. Transcriptomic analysis showed that *LDHC*-silenced HCC-1954 cells exhibited the activation of STAT3 signaling, suggesting that HCC-1954 cells can compensate for the detrimental effects of LDHC depletion through the STAT3 pro-survival pathway. Indeed, the inhibition of STAT3 following *LDHC* silencing restored the loss-of-tumor cell survival. Furthermore, the treatment of HCC-1954 cells with a STAT3 inhibitor alone significantly decreased short-term and long-term cancer cell survival, indicating that these cells may benefit from single-agent treatment with STAT3 inhibitors (Figure 5). In line with this, previous reports have indicated a potential benefit of STAT3 inhibition in Her2-enriched breast cancer, although clinical validation is still lacking [19,20]. Further to this, additional Her2-enriched cell lines need to be used to validate our current findings.

STAT3 is a core member of the STAT family of transcription factors, which play important roles in various cellular processes including proliferation, differentiation, apoptosis, angiogenesis and inflammation [21]. STAT3 is strongly associated with tumor development and progression. The hyperactivation of the STAT3 signaling pathway has been detected in a multitude of cancer types, where it has been associated with poor clinical outcomes [21]. Thus, numerous clinical trials are currently investigating the efficacy and safety of several STAT3 inhibitors [22]. It is known to act as an oncogene through gain-of-function mutations in hematological malignancies [23,24], and it promotes pro-tumorigenic biological processes such as proliferation, migration, invasion, and cell survival [25,26]. Furthermore, STAT3 plays a critical role in regulating the DNA damage response pathway by modulating both the ATM-Chk2 and ATR-Chk1 pathways, whereby the loss of STAT3 renders cells more sensitive to DNA damage [27]. In accordance, the inhibition of STAT3 sensitizes cancer cells to radiation- and oxidative stress-induced DNA damage [28,29]. Hence, we speculate that *LDHC* silencing differentially regulates STAT3 signaling depending on the cell line’s genetic context, resulting in divergent responses to DNA damage. Several studies have suggested that phosphorylation at the serine 727 (S727) site of STAT3 is required for the maximal activation of the signaling pathway, involving different kinases such as PKC delta, ERK, JNK, MAPK and mTOR [30,31,32,33,34,35,36]. Of note, *LDHC*-silenced HCC-1954 cells showed an increase in S727 STAT3 phosphorylation, reflecting a strong induction of STAT3 signaling. Molecularly, it remains to be determined whether the metabolic function of LDHC can facilitate its pro-survival effects on STAT3 signaling. It is likely that when LDHC expression is reduced, pyruvate levels increase. Consequently, the synthesis of diacylglycerol would increase via the TCA cycle and free fatty acid biosynthesis pathway, ultimately leading to the activation of STAT3. This mechanism may be of particular importance in tumors with elevated de novo lipogenesis such as Her2-enriched tumors, which also display a positive feedback loop between Her2 and the lipogenic enzyme FASN [35,36]. Future studies are needed to decipher the impact of *LDHC* silencing on metabolic pathways in tumor cells and how these can be exploited in cancer therapy. Furthermore, whether LDHC-targeted therapy, either as monotherapy or combination therapy, could be beneficial to patients with other breast cancer subtypes remains to be determined. Finally, these findings need to be validated using in vivo xenograft models. 

## 5. Conclusions

Our findings demonstrate that STAT3 signaling may impact the therapeutic potential of targeting LDHC in a subtype-dependent context. LDHC targeting demonstrates a beneficial response, particularly in basal-like breast cancer cellular models, through the inhibition of the LDHC-STAT3 axis.

## Data Availability

The RNAseq data generated in this study have been submitted to NCBI BioProject (http://www.ncbi.nlm.nih.gov/bioproject) under BioProject number PRJNA1020744.

## Supplementary Materials

The following supporting information can be downloaded at: https://www.mdpi.com/article/10.3390/cancers16132451/s1. Table S1. Differentially expressed genes in BT-549 cells following *LDHC* silencing. Table S2. Differentially expressed genes in HCC-1954 cells following *LDHC* silencing. Figure S1. Densitometry quantification of Western blotting. File S1: Western blot information.
