# Supplementary material for: The LDHC-STAT3 Signaling Network Is a Key Regulator of Basal-like Breast Cancer Cell Survival
Source: Cancers (Basel). 2024 Jul 4;16(13):2451. doi: 10.3390/cancers16132451 (PMC11240808; doi:10.3390/cancers16132451)
Supplement: Supplementary file 1 [file cancers-16-02451-s001.zip › cancers-2788160-supplementary/File S1.pdf]

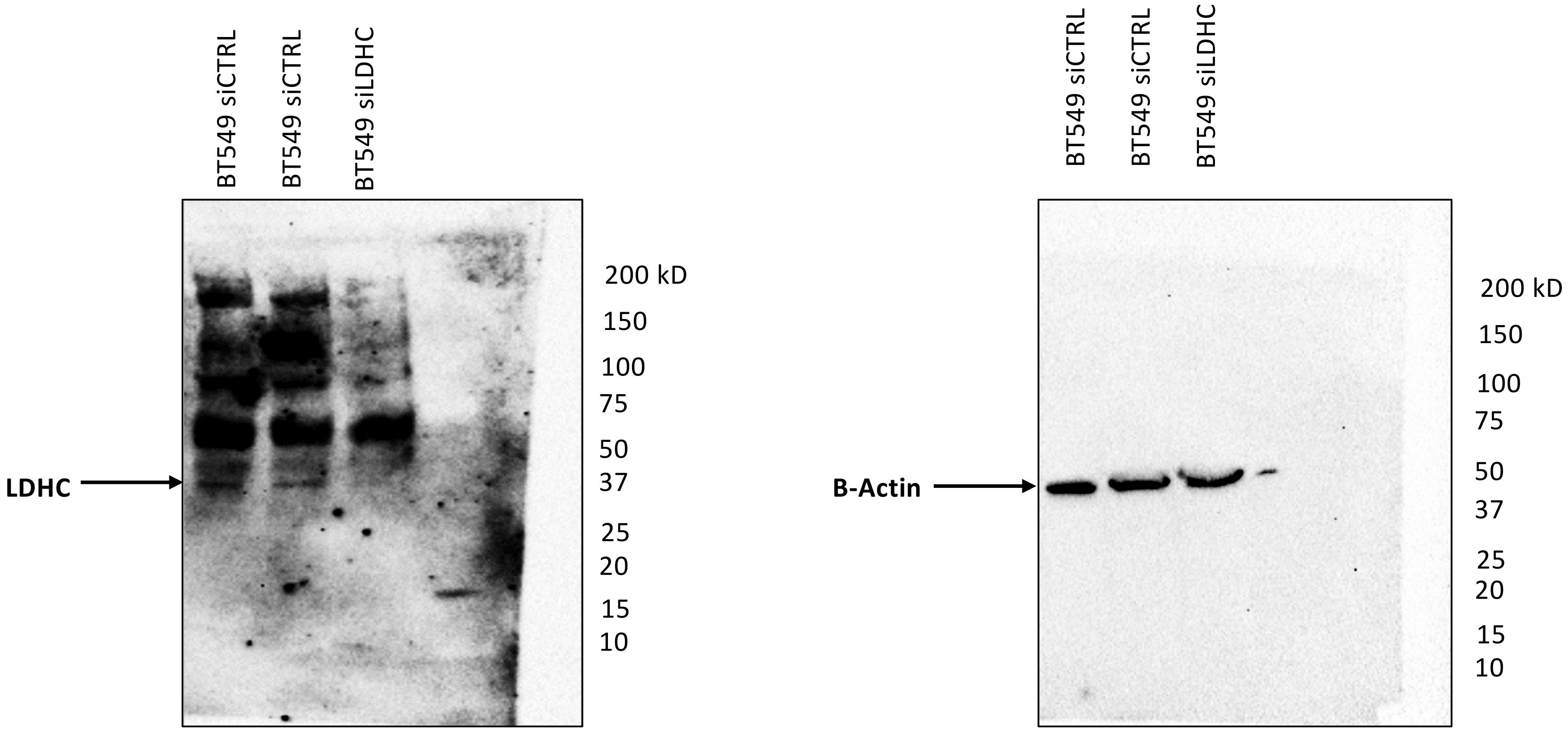
BT549 siCTRL
BT549 siCTRL
BT549 siLDHC
200 kD
150
100
75
50
37
25
20
15
10
LDHC
BT549 siCTRL
BT549 siCTRL
BT549 siLDHC
200 kD
150
100
75
50
37
25
20
15
10
B-Actin

FIGURE 2A

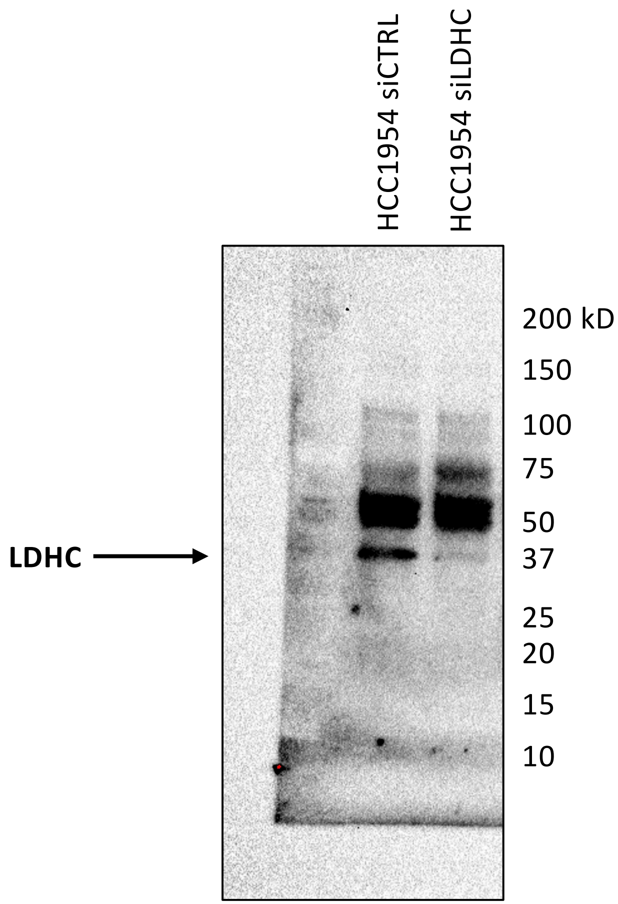
HCC1954 siCTRL
HCC1954 siLDHC
200 kD
150
100
75
50
37
25
20
15
10
LDHC

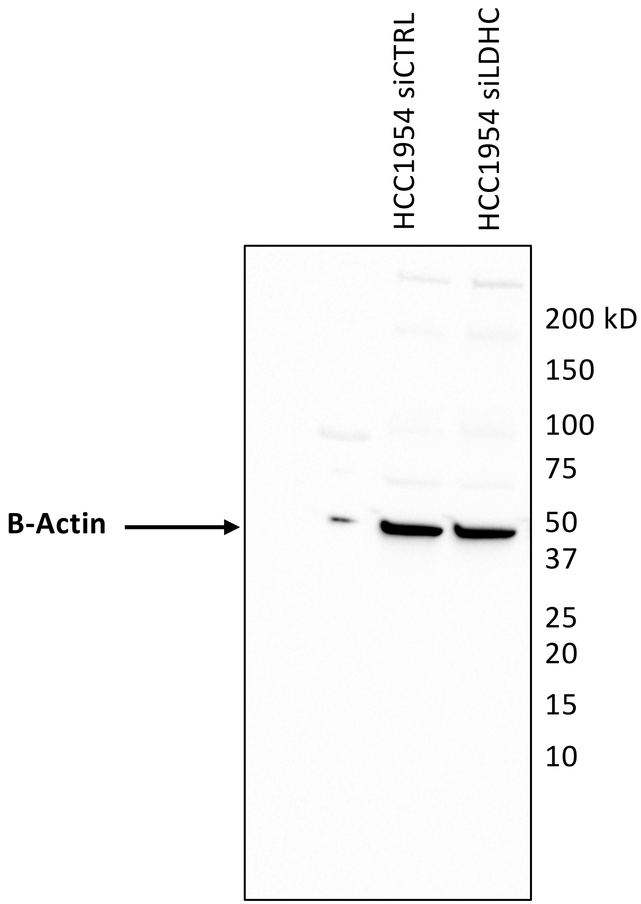
HCC1954 siCTRL
HCC1954 siLDHC
200 kD
150
100
75
50
37
25
20
15
10
B-Actin

FIGURE 2A

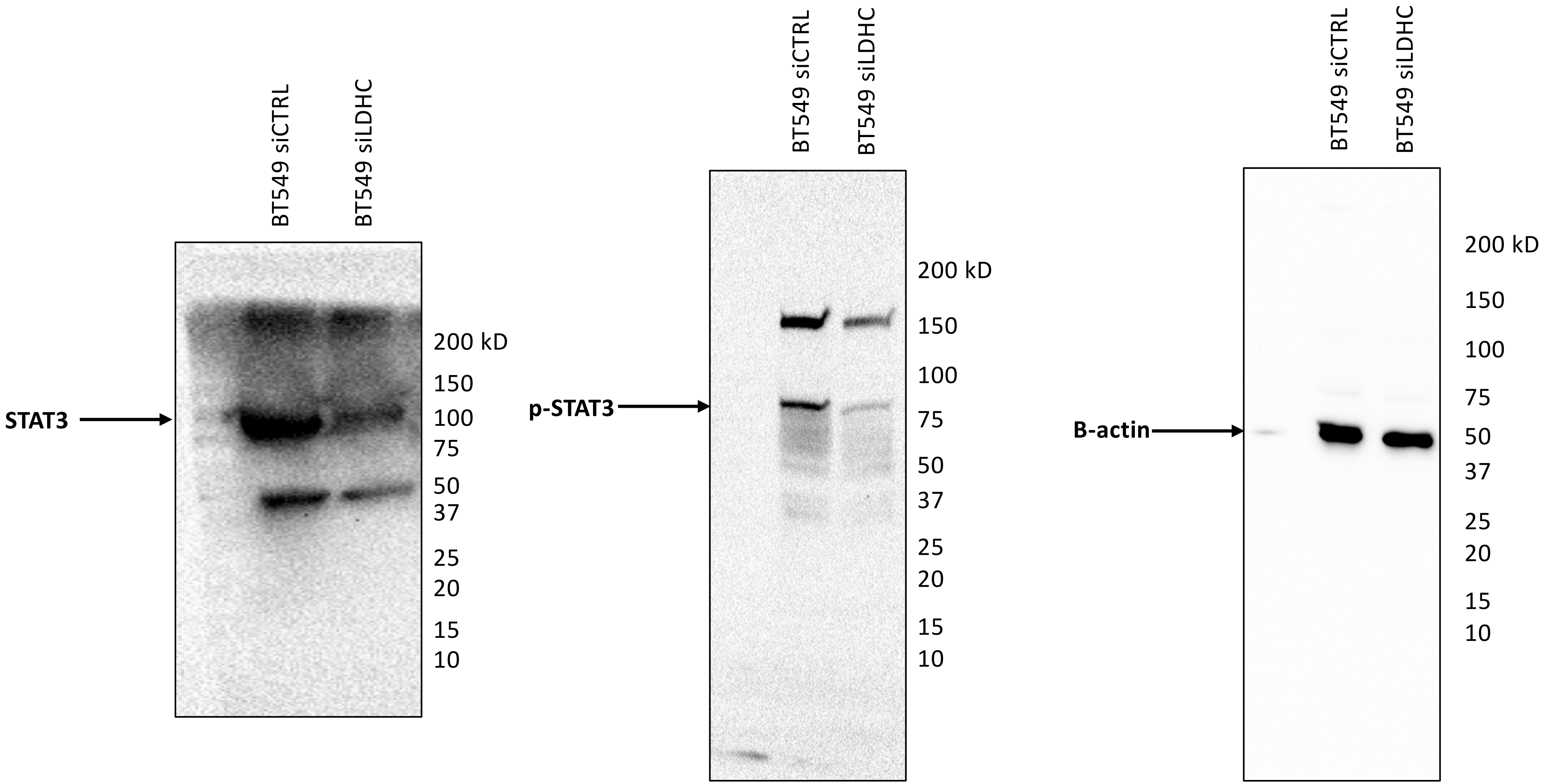
BT549 siCTRL
BT549 siLDHC
STAT3
200 kD
150
100
75
50
37
25
20
15
10
BT549 siCTRL
BT549 siLDHC
p-STAT3
200 kD
150
100
75
50
37
25
20
15
10
BT549 siCTRL
BT549 siLDHC
B-actin
200 kD
150
100
75
50
37
25
20
15
10

FIGURE 3A

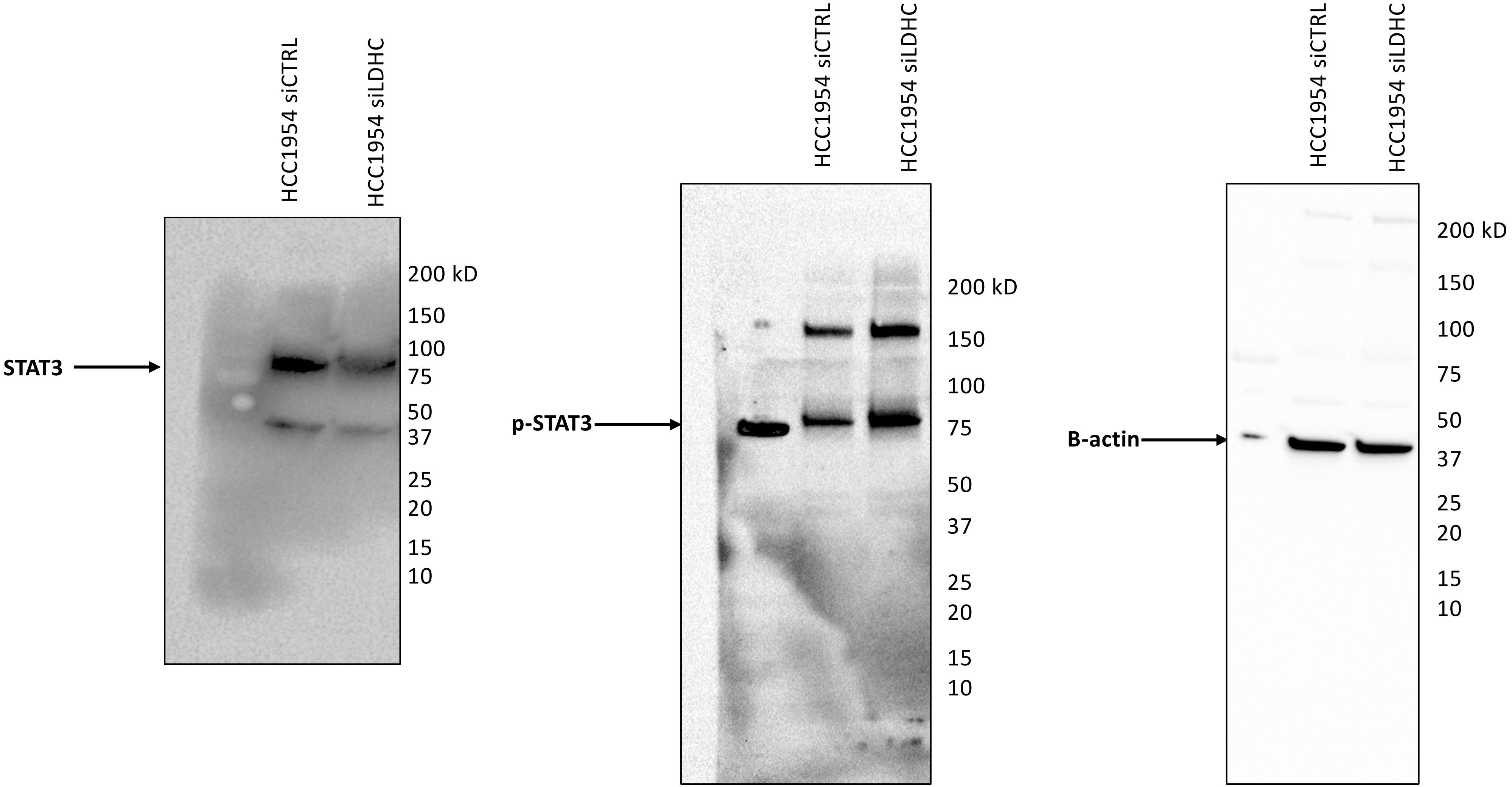

HCC1954 siCTRL
HCC1954 siLDHC
STAT3
200 kD
150
100
75
50
37
25
20
15
10
p-STAT3
B-actin

FIGURE 3A

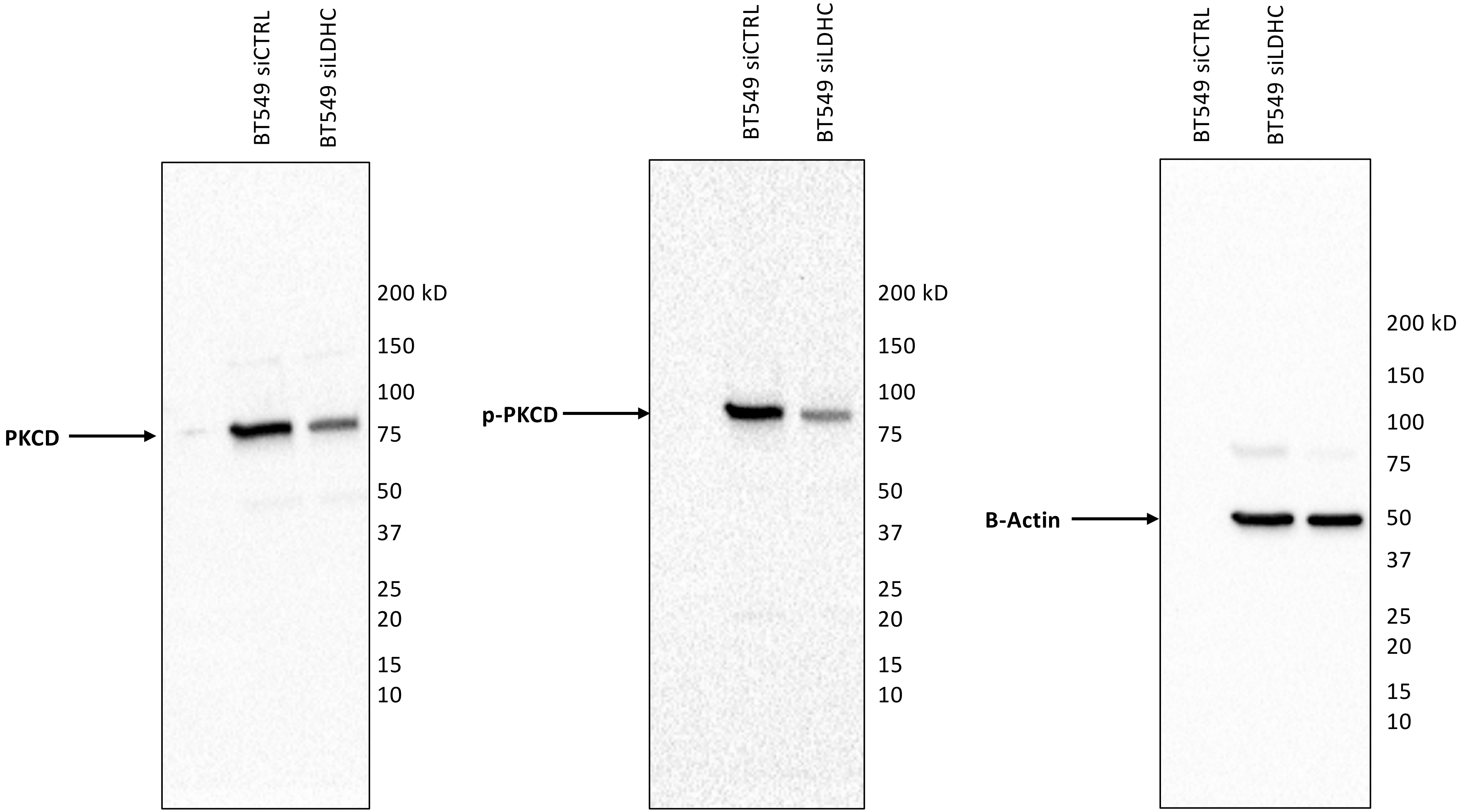
BT549 siCTRL
BT549 siLDHC
PKCD
200 kD
150
100
75
50
37
25
20
15
10
BT549 siCTRL
BT549 siLDHC
p-PKCD
200 kD
150
100
75
50
37
25
20
15
10
BT549 siCTRL
BT549 siLDHC
B-Actin
200 kD
150
100
75
50
37
25
20
15
10

FIGURE 3A

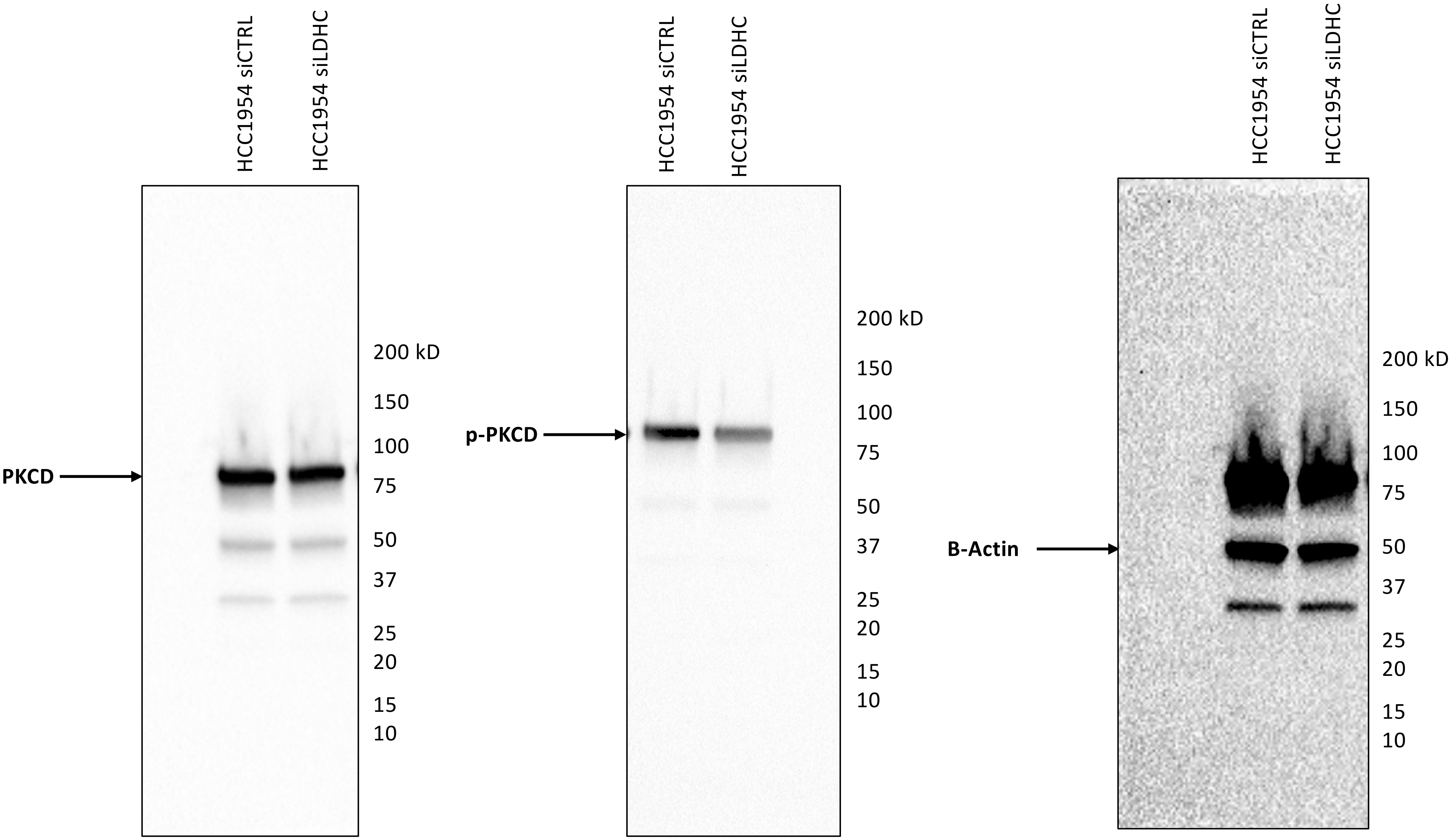
HCC1954 siCTRL
HCC1954 siLDHC
PKCD
200 kD
150
100
75
50
37
25
20
15
10
HCC1954 siCTRL
HCC1954 siLDHC
p-PKCD
200 kD
150
100
75
50
37
25
20
15
10
HCC1954 siCTRL
HCC1954 siLDHC
B-Actin
200 kD
150
100
75
50
37
25
20
15
10

FIGURE 3A

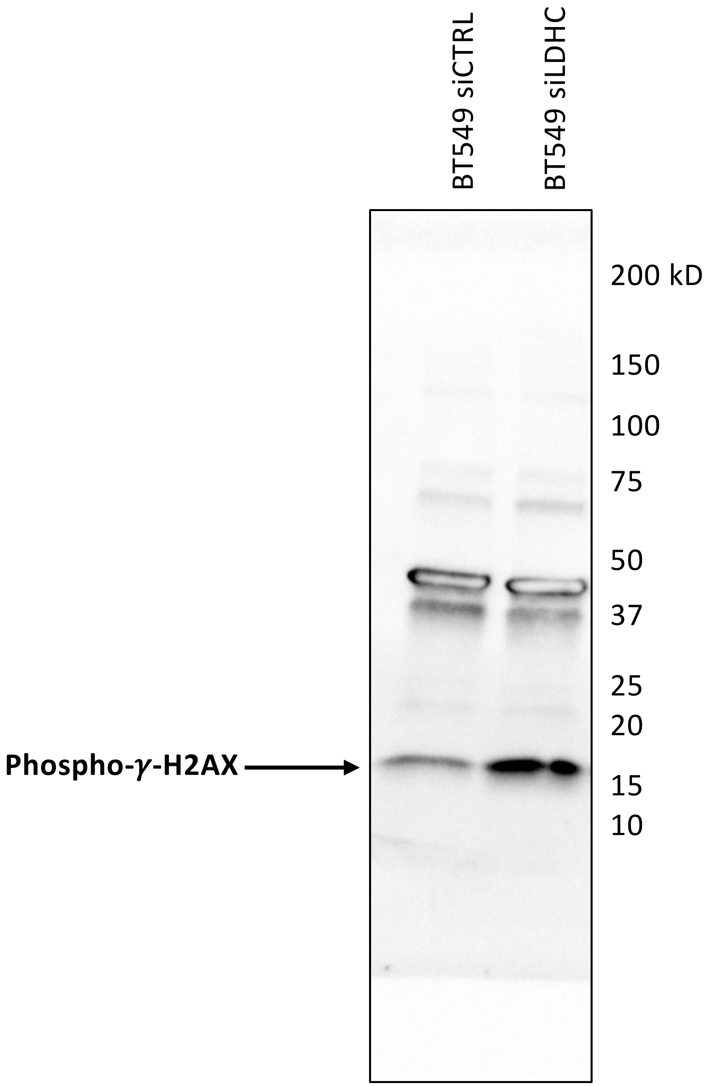
BT549 siCTRL
BT549 siLDHC
200 kD
150
100
75
50
37
25
20
15
10
Phospho-γ-H2AX

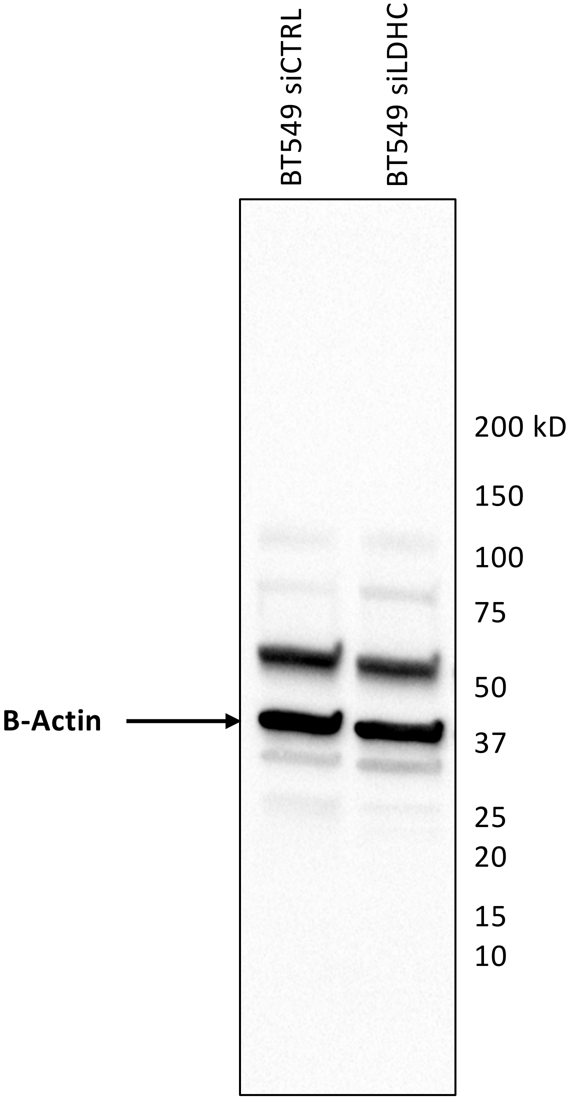
BT549 siCTRL
BT549 siLDHC
200 kD
150
100
75
50
37
25
20
15
10
B-Actin

FIGURE 3B

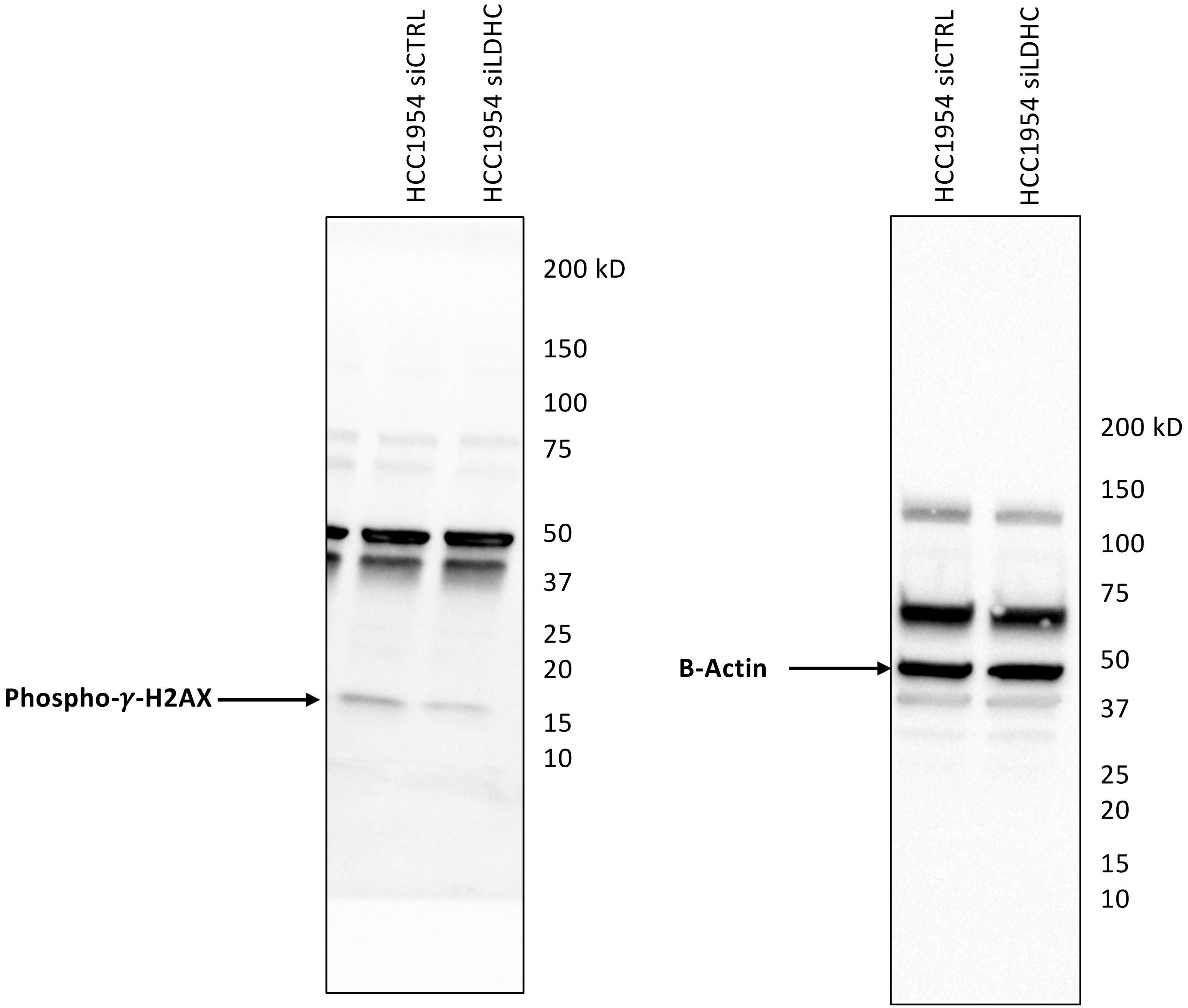

HCC1954 siCTRL
HCC1954 siLDHC
200 kD
150
100
75
50
37
25
20
15
10
Phospho-γ-H2AX
HCC1954 siCTRL
HCC1954 siLDHC
200 kD
150
100
75
50
37
25
20
15
10
B-Actin

FIGURE 3B

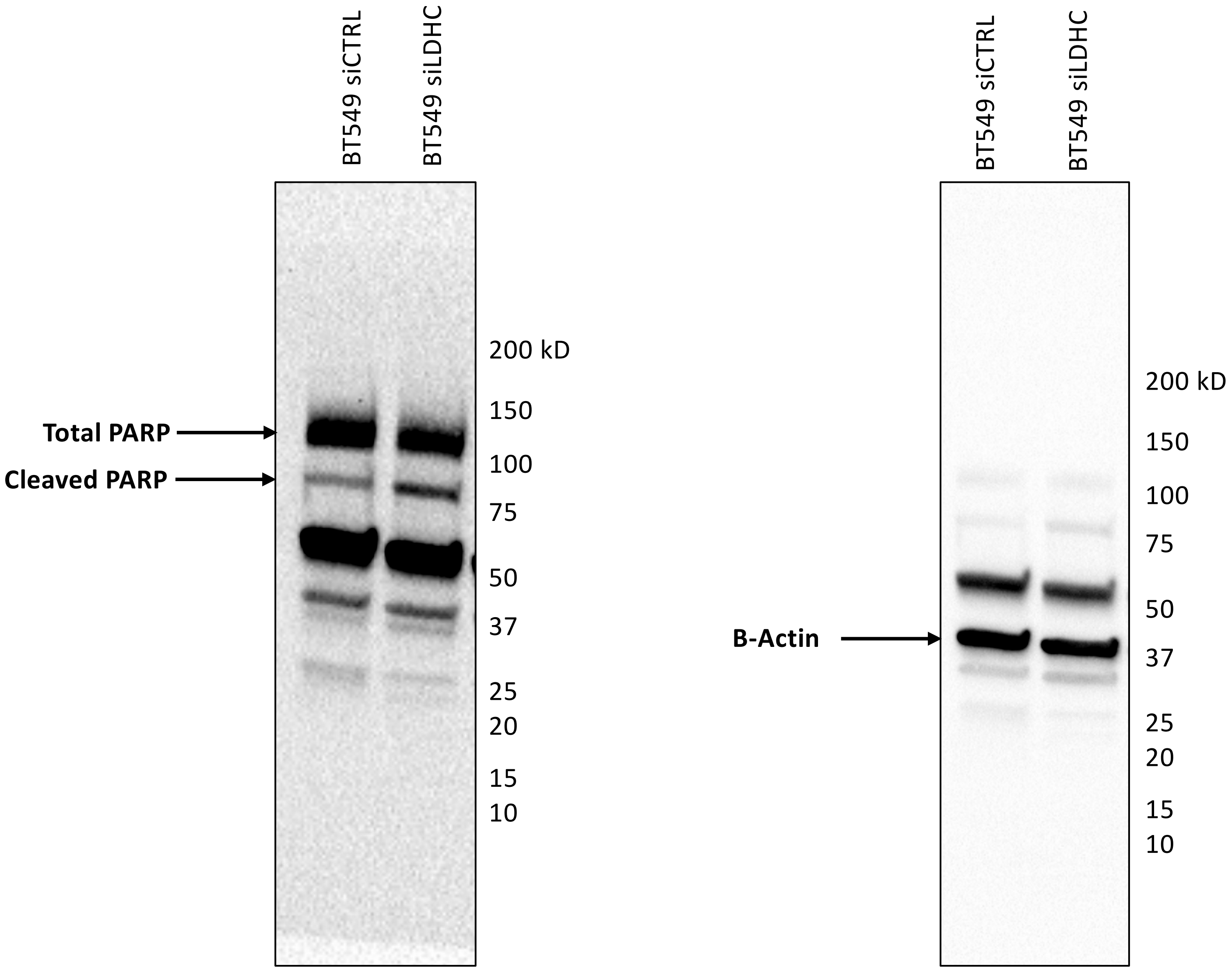
BT549 siCTRL
BT549 siLDHC
Total PARP
Cleaved PARP
200 kD
150
100
75
50
37
25
20
15
10
BT549 siCTRL
BT549 siLDHC
B-Actin
200 kD
150
100
75
50
37
25
20
15
10

FIGURE 3B

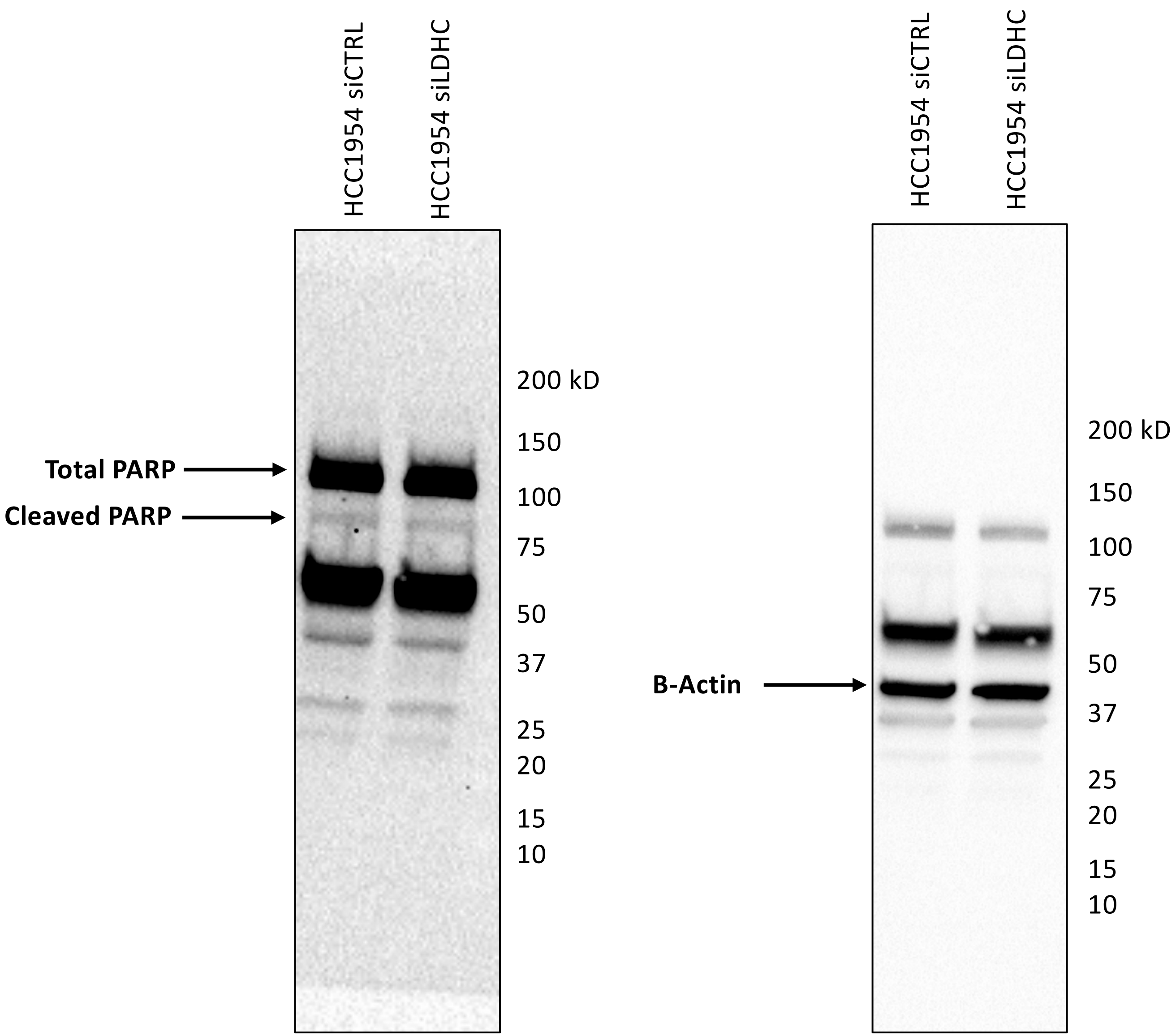
HCC1954 siCTRL
HCC1954 siLDHC
Total PARP
Cleaved PARP
200 kD
150
100
75
50
37
25
20
15
10
HCC1954 siCTRL
HCC1954 siLDHC
B-Actin
200 kD
150
100
75
50
37
25
20
15
10

FIGURE 3B

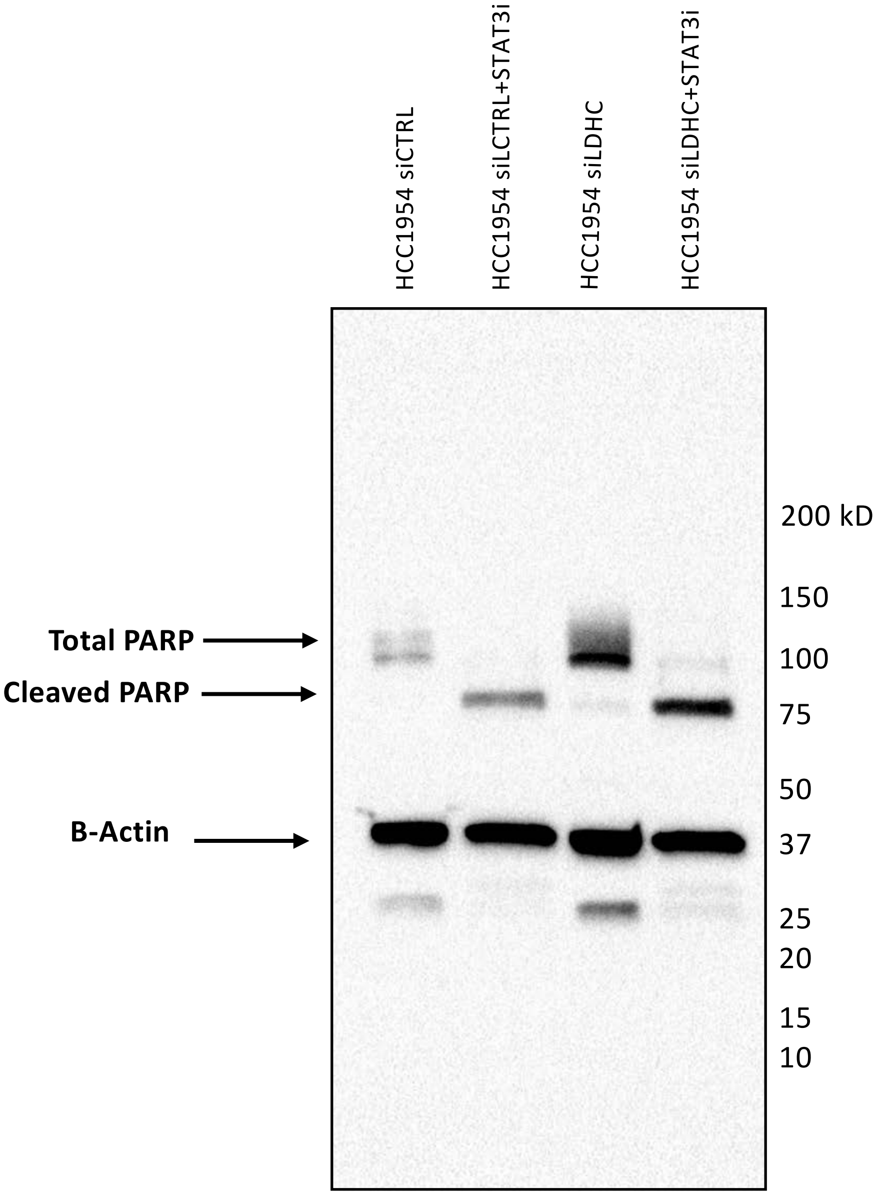
HCC1954 siCTRL
HCC1954 siLCTRL+STAT3i
HCC1954 siLDHC
HCC1954 siLDHC+STAT3i
Total PARP
Cleaved PARP
B-Actin
200 kD
150
100
75
50
37
25
20
15
10

FIGURE 4A

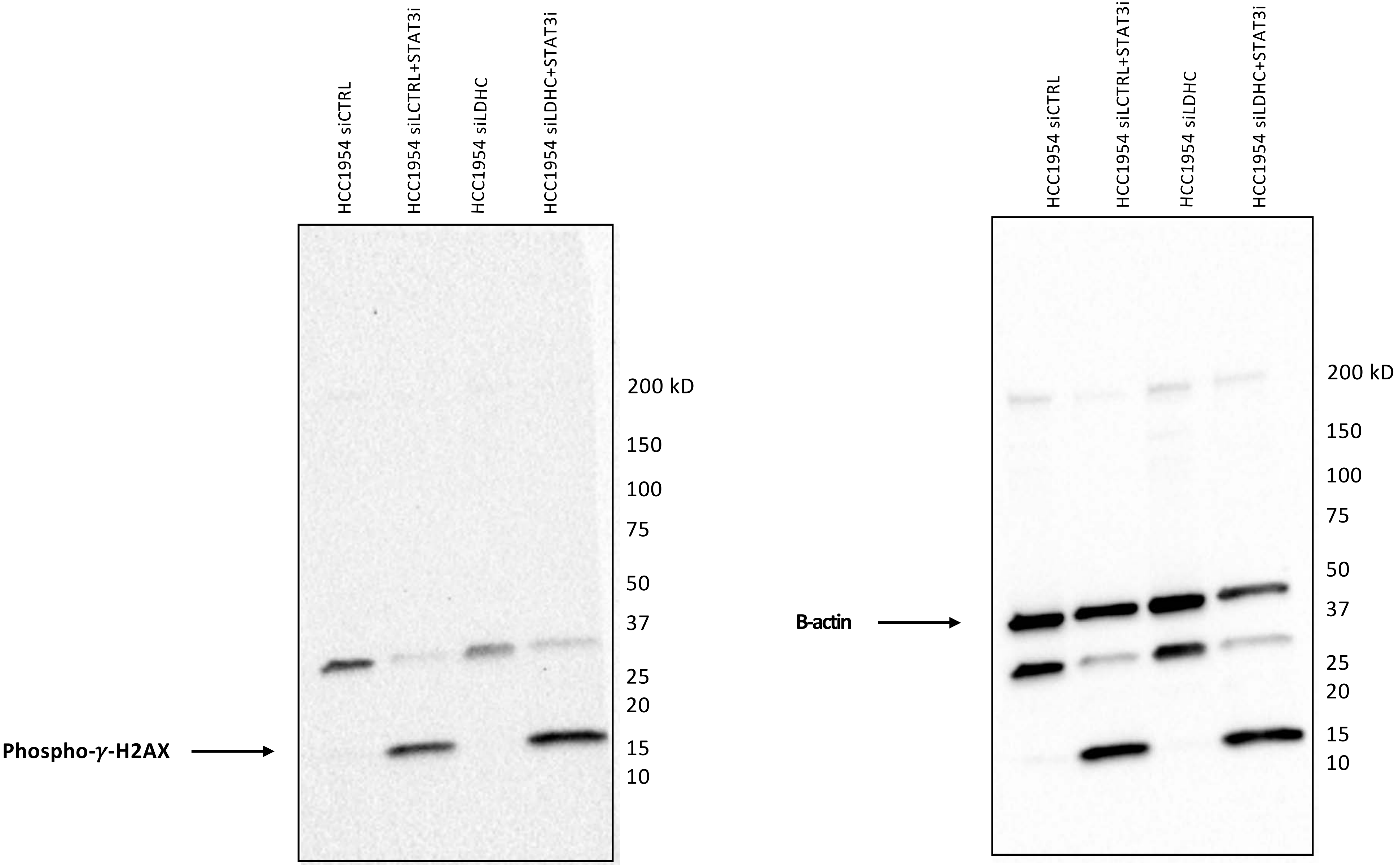
HCC1954 siCTRL
HCC1954 siLCTRL+STAT3i
HCC1954 siLDHC
HCC1954 siLDHC+STAT3i
200 kD
150
100
75
50
37
25
20
15
10
Phospho-γ-H2AX
HCC1954 siCTRL
HCC1954 siLCTRL+STAT3i
HCC1954 siLDHC
HCC1954 siLDHC+STAT3i
200 kD
150
100
75
50
37
25
20
15
10
B-actin

FIGURE 4A
