# Supplementary figures and images for: The LDHC-STAT3 Signaling Network Is a Key Regulator of Basal-like Breast Cancer Cell Survival
Source: Cancers (Basel). 2024 Jul 4;16(13):2451. doi: 10.3390/cancers16132451 (PMC11240808; doi:10.3390/cancers16132451)

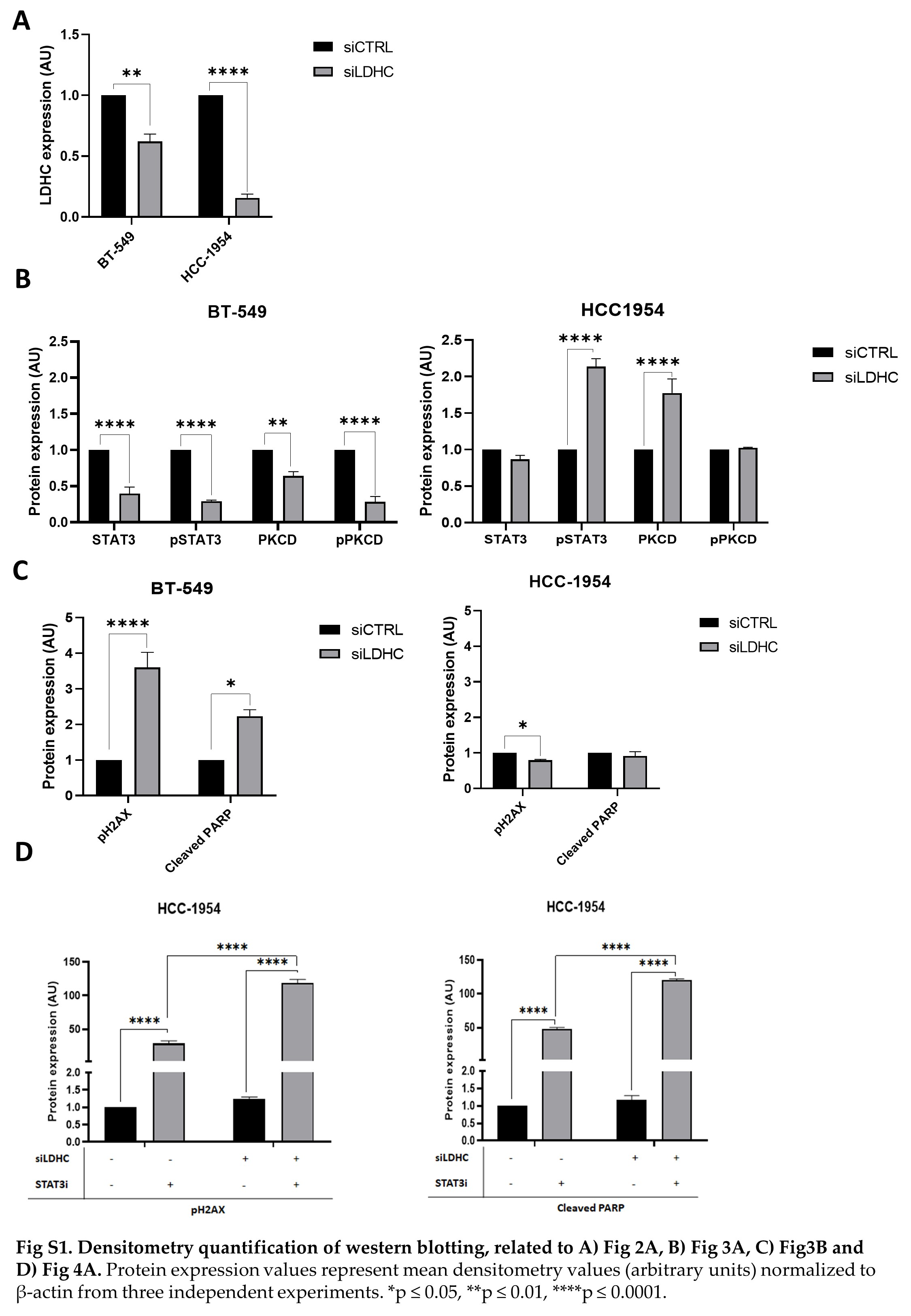

Supplement: Supplementary file 1 [file cancers-16-02451-s001.zip › cancers-2788160-supplementary/Figure S1.tiff]
